# Spotted fever group rickettsiae (SFGR) detection in ticks following reported human case of Japanese spotted fever in Niigata Prefecture, Japan

**DOI:** 10.1038/s41598-021-81587-9

**Published:** 2021-01-28

**Authors:** Reiko Arai, Megumi Sato, Miwako Kato, Junko Aoki, Akiko Nishida, Kaori Watanabe, Chika Hirokawa, Sumire Ikeda, Kozo Watanabe, Maria Angenica F. Regilme, Marcello Otake Sato, Tsutomu Tamura

**Affiliations:** 1 Niigata Prefectural Institute of Public Health and Environmental Sciences, 314-1 Sowa, Nishi-ku, Niigata, 950-2144 Japan; 2 Graduate School of Health Sciences, Niigata University, 2-746 Asahimachi-dori, Chuo-ku, Niigata, 951-8518 Japan; 3 Department of Civil and Environmental Engineering, Faculty of Engineering, Ehime University, Bunkyo-cho 3, Matsuyama, Ehime 790-8577 Japan; 4 Department of Tropical Medicine and Parasitology, Dokkyo Medical University, 880 Kitakobayashi, Mibu-machi, Shimotsuga-gun, Tochigi, 321-0293 Japan

**Keywords:** Microbiology, Infectious diseases, Epidemiology

## Abstract

Japanese spotted fever, a tick-borne disease caused by *Rickettsia japonica*, was firstly described in southwestern Japan. There was a suspicion of *Rickettsia japonica* infected ticks reaching the non-endemic Niigata Prefecture after a confirmed case of Japanese spotted fever in July 2014. Therefore, from 2015 to 2017, 38 sites were surveyed and rickettsial pathogens were investigated in ticks from north to south of Niigata Prefecture including Sado island. A total of 3336 ticks were collected and identified revealing ticks of three genera and ten species: *Dermacentor taiwanensis*, *Haemaphysalis flava*, *Haemaphysalis hystricis*, *Haemaphysalis longicornis*, *Haemaphysalis megaspinosa*, *Ixodes columnae*, *Ixodes monospinosus*, *Ixodes nipponensis*, *Ixodes ovatus,* and *Ixodes persulcatus*. Investigation of rickettsial DNA showed no ticks infected by *R. japonica*. However, three species of spotted fever group rickettsiae (SFGR) were found in ticks, *R. asiatica*, *R. helvetica,* and *R. monacensis*, confirming Niigata Prefecture as a new endemic area to SFGR. These results highlight the need for public awareness of the occurrence of this tick-borne disease, which necessitates the establishment of public health initiatives to mitigate its spread.

## Introduction

Japanese spotted fever is a tick-borne disease caused by *Rickettsia japonica*; the disease was first described in Tokushima Prefecture in southwestern Japan and named by Mahara (1989; 1985)^1^. Clinically, the major complaints are fever after 2 to 8 days of tick bite and rash. In Japan, approximately 250 cases are reported mainly in the western area of Japan, and 16 deaths cases were reported for ten years from 2007 to 2016^2^. With a wide spectrum of host ticks, *R. japonica* has been detected in eight species of ticks within three genera (*Haemaphysalis*, *Dermacentor and Ixodes*)^3^. Moreover, in recently described cases of Spotted Fever Group Rickettsiae (SFGR), the disease is caused by species other than *R. japonica*^3,4^.

The first cases of Japanese spotted fever in the northern part of the coastal area of the Sea of Japan were reported in 2014, in Fukui and Niigata Prefectures^4,5^. After an epidemiological investigation on the confirmed case of *Rickettsia japonica* occurred in Niigata Prefecture, the most probable place of contact with ticks was near the patient’s house in an urban area despite no ticks were collected for identification. The last tick survey in Niigata Prefecture occurred in the ’50s^6^, and it is unknown if there is a change in the endemic ticks’ species in the area. Therefore, there is a concern about the species and the habitat of the ticks harboring *R. japonica* in Niigata Prefecture which could spread the disease. This study was conducted to detect the tick prevalence and SFGR prevalence by species of ticks in Niigata Prefecture after the occurrence of a Japanese spotted fever human case.

## Methods

### Area of the study and collection of ticks

**Figure 1:**
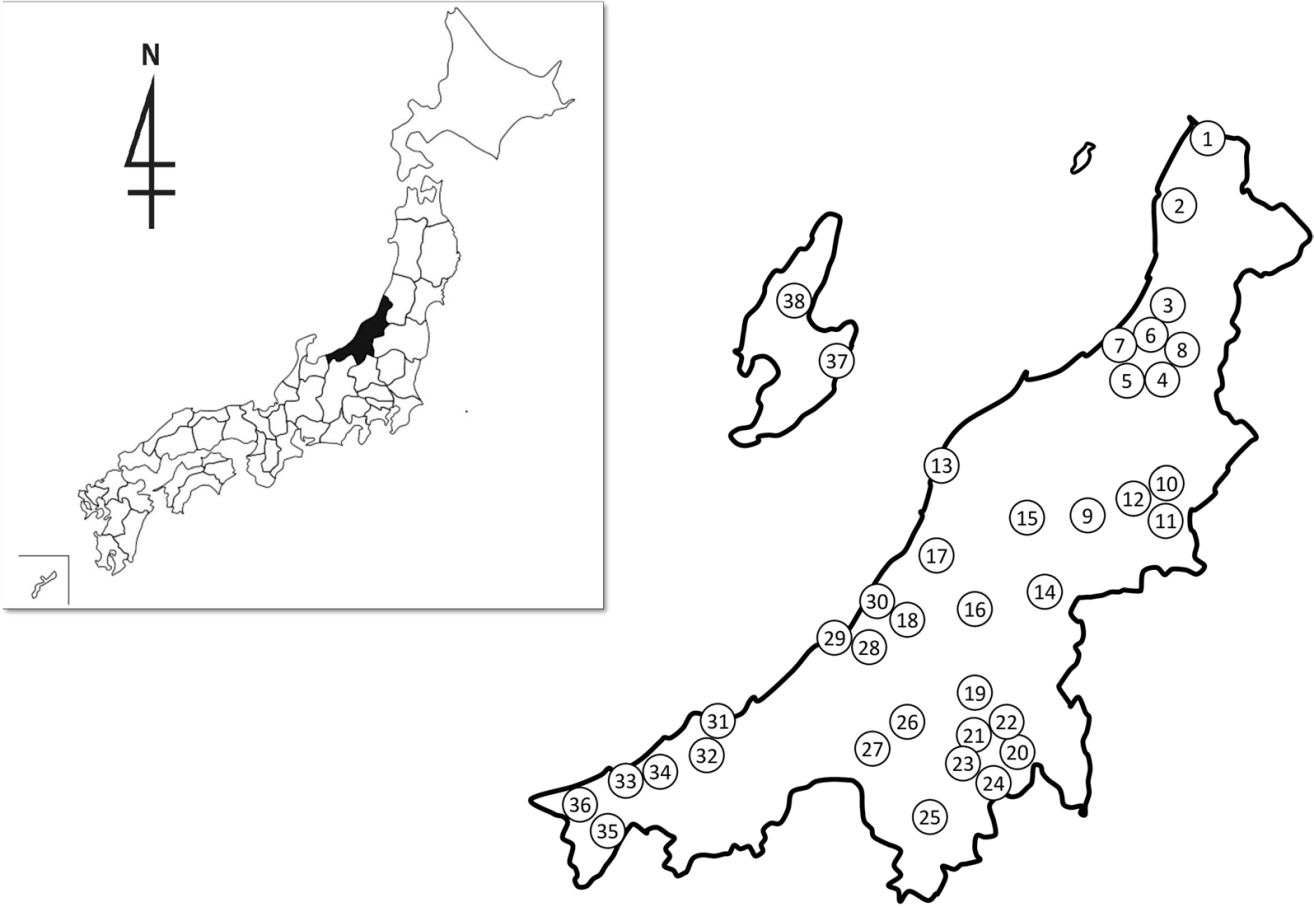
Tick collection sites in Niigata Prefecture. On the left the localization of Niigata Prefecture in Japan. On the right, the numbers indicate the 38 tick collection sites covering the 4 geographical areas of Niigata Prefecture: Northern (Kaetsu), Central (Chuetsu) and Southern (Joetsu) areas of Niigata Prefecture corresponds to the collection sites 1 to 13, 14 to 30, 31 to 36, respectively. Sado Island area corresponds to the collection sites 37 and 38. Maps created using the Geographical Survey Institute Map Vector from the Geospatial Information Authority of Japan (https://maps.gsi.go.jp/).

The ticks were collected by flagging method in 38 sites from north to south of Niigata Prefecture including Sado island from June 2015 to November 2017, completing a total of 77 field surveys (Table 1, Fig. 1). Collection sites where humans were likely to be exposed to ticks such as parks, forests with hiking courses, and camping areas were chosen for sampling.

**Table 1:** Tick collection sites and elevation in Niigata Prefecture. The Northern (Kaetsu), Central (Chuetsu) and Southern (Joetsu) areas of Niigata Prefecture corresponds to the collection sites 1 to 13, 14 to 30, and 31 to 36, respectively. Sado Island area corresponds to the collection sites 37 and 38.

No	Site	Elevation (m)
1	Mt. Nihonkoku	179–300
2	Mt. Shinbodake	560
3	Kawabe	20
4	Ohmineyama Park	80
5	Ijimino Park	20
6	Shiratori Park	190–220
7	Sekizawa Forest Park	130
8	Tainaidaira Campground	130
9	Sugikawa Park	130
10	Akasakiyama Forest Park	330
11	Kirinzan Park	100
12	Aganogawa Line Naturel Park	130
13	Mt. Kakuda	140–250
14	Yoshigahira Natural Park	410
15	Gejougawa Dam Park	40
16	Happoudai ikoi-no-mori	840
17	Umamichi Forest Park	400
18	Masugatayama Natural Park	550
19	Tsukioka Park	420
20	Mt. Hakkai	1200–1300
21	Ohsaki Dam Park	500
22	Okura Forest Park	540
23	Sakado Castle Ruins	280–320
24	Ikazawa Campground	600
25	Yuzawa Kogen	840–950
26	Niroku Park	580
27	Bijinbayashi Forest	310
28	Satogaike Sports Field	20
29	Kujiranami	5
30	Akata Castle Ruins	50
31	Tanihama	230
32	Kuwadori Forest park	360–450
33	Takanomine Plateau Forest Park	230
34	Mt. Fudo	650
35	Takanamigaike Campground	700
36	Mt. Myojo	430
37	Sugiike Pond	720
38	Mt. Donden	700–930

### Ticks identification

Collected ticks were identified morphologically under stereoscope based on the key by Yamaguti^7^ and separated by the species, sex and growth stages, collection day and the collection sites. The ticks were separated in micro tubes and stored at − 80 °C until further processing. The identification of ticks with insufficient morphologic characteristics was confirmed by DNA sequencing of the mitochondrial 16S rDNA gene, as previously described^8^ (data not shown).

### DNA extraction

The DNA extraction and purification were done individually for ticks in the adult stage. For ticks in larvae or nymph stages, the DNA was extracted individually or from a pool of 2 to 5 individuals. Ticks were thawed and homogenized using a cell crasher (FastPrep-24, M. P. Biomedicals) in tubes with six steel beads of 3 mm diameter (Metal Bead Lysing Matrix, M. P. Biomedicals). DNA was purified using High Pure PCR Template Preparation Kit (Roche) according to the manufacturer’s instruction and stored at -80 °C until further processing.

### PCR and sequencing analyses

To detect *Rickettsia* species, nested-PCR for the genus-common 17-kDa antigen gene (17-kDa), citrate synthase gene (*gltA*), spotted fever group (SFG)-specific outer membrane protein A gene (*rOmpA*) and outer membrane protein B gene (*rOmpB*) were targeted. Firstly, the nested PCR targeting 17-kDa protein, and positive samples were tested with another nested PCR targeting *gltA*; samples that were positive with both PCR assays were concluded as SFGR positive. Additionally, two other nested PCR assays targeting *rOmpA* and *rOmpB* were conducted, and the positive samples for four nested PCR were sequenced, and the *Rickettsia* spp. were identified.

The PCR primers used in this study are summarized in Table 2^9–13^. PCR amplicons were purified using AMPure XP (Beckman Coulter Co., Japan) and sequenced directly using a Big Dye Terminator Cycle Sequence Kit (Applied Biosystems, USA) and Applied Biosystems 3500 Genetic Analyzer. The analyses of the obtained sequences were carried out using MEGA 5.2^14^. The obtained sequences from this study and from DDBJ/EMBL/GenBank databases were aligned by Clustal W 2.0. Neighbor-joining phylogenetic tree construction and bootstrap analysis (1000 replicates) were performed according to the Kimura 2-parameter distances method.

**Table 2:** Primer pairs used for SFGR detection and typing.

Primer		Nucleotide sequence (5′-3′)	Target gene (amplicon size)	References
1st	Rr17k.1p (forward)	TTTACAAAATTCTAAAAACCAT	17 kDa (450 bp)	^9^
	Rr17k.539n (reverse)	TCAATTCACAACTTGCCATT		
2nd	Rr17k.90p (forward)	GCTCTTGCAACTTCTATGTT		
	Rr17k.539n (reverse)	TCAATTCACAACTTGCCATT		
1st	RpCs.780p (forward)	GACCATGAGCAGAATGCTTCT	*gltA *(382 bp)	^9,10^
	RpCs.1258n (reverse)	ATTGCAAAAAGTACAGTGAAC		
2nd	RpCs.877p (forward)	GGGGGCCTGCTCACGGCGG		
	RpCs.1258n (reverse)	ATTGCAAAAAGTACAGTGAAC		
1st	RR 190–70 (forward)	ATGGCGAATATTTCTCCAAAA	*rOmpA *(540 bp)	^11,12^
	RR 190–701(reverse)	GTTCCGTTAATGGCAGCATCT		
2nd	190-FN1 (forward)	AAGCAATACAACAAGGTC		
	190-RN1 (reverse)	TGACAGTTATTATACCTC		
1st	rompB OF (forward)	GTAACCGGAAGTAATCGTTTCGTAA	*rOmpB *(426 bp)	^13^
	rompB OR (reverse)	GCTTTATAACCAGCTAAACCACC		
2nd	rompB SFG IF (forward)	GTTTAATACGTGCTGCTAACCAA		
	rompB SFG/TG IR (reverse)	GGTTTGGCCCATATACCATAAG		

### Ethics approval and consent to participate

No ethical permissions were necessary for this study as the parasites were collected from the environment of public places.

## Results

### Tick species identification

A total of 3336 tick specimens were collected from the 38 sites in Niigata Prefecture (Fig. 1). 3308 ticks were identified to ten species under three genera, while 28 ticks could only be identified as *Haemaphysalis* spp. (Table 3). The highest frequency was obtained for *Haemaphysalis longicornis*, collected from 33 of 38 sites, followed by *Haemaphysalis flava* from 24 sites, and *Ixodes ovatus* collected from 31 sites. These three species comprised 96.2% of all the collected ticks in this study. Other tick species collected were *Ixodes nipponensis* collected from 14 sites, *Dermacentor taiwanensis* from seven sites, *Ixodes monospinosus* from nine sites, *Ixodes persulcatus* from five sites, *Haemaphysalis megaspinosa* from three sites, *Ixodes columnae* from two sites, and *Haemaphysalis hystricis* from one site (No. 13).

**Table 3:** Prevalence of rickettsial genes detected from ticks by PCR.

Tick species	No. positive/tested						
	Adult				Nymph^a^	Larva^a^	Total
	Female	Male	Subtotal	%			
*D. taiwanensis*	0/14	0/15	0/29	0.0	0/1	–	0/30
*H. flava*	0/145	2/161	2/306	0.7	9/202	1/31	12/539
*H. hystricis*	0/2		0/2	0.0	0/1	–	0/3
*H. longicornis*	0/40	0/10	0/50	0.0	6/232	3/104	9/386
*H. megaspinosa*	0/2	0/1	0/3	0.0	–	–	0/3
*Haemaphysalis* spp.	0/1	0/1	0/2	0.0	0/1	0/11	0/14
*I. columnae*	–	–	–	–	–	0/2	0/2
*I. monospinosus*	3/11	1/1	4/12	33.3	0/1	0/1	4/14
*I. nipponensis*	2/4	5/12	7/16	43.8	7/10	0/2	14/28
*I. ovatus*	17/177	12/167	29/344	8.4	0/2	–	29/346
*I. persulcatus*	0/2	0/5	0/7	0.0	0/1	–	0/8
Total	22/398	20/373	42/771	5.4	22/451	4/151	68/1373

^a^Shown by no. pool (2 to 5 ticks/sample).

### SFGR prevalence in collected ticks

From 1373 DNA samples of a total of 38 collection sites, 68 samples from 20 sites were positive for SFGR. The tick species presenting SFGR were: *H. flava, H. longicornis, I. monospinosus, I. nipponensis*, and *I. ovatus*. Overall, the *Rickettsia* detection rate for all tested samples adult stage ticks was 5.4%. SFGR positivity by species of ticks were 0.7% in *H. flava*, 8.4% in *I. ovatus*, 33.3% in *I. monospinosus* and 43.8% in *I. nipponensis* (Table 3). For *H. longicornis*, the positives samples in SFGR were from nymph and larval stage, but not from adult stage ticks.

### SFGR species identification and geographical prevalence

**Figure 2:**
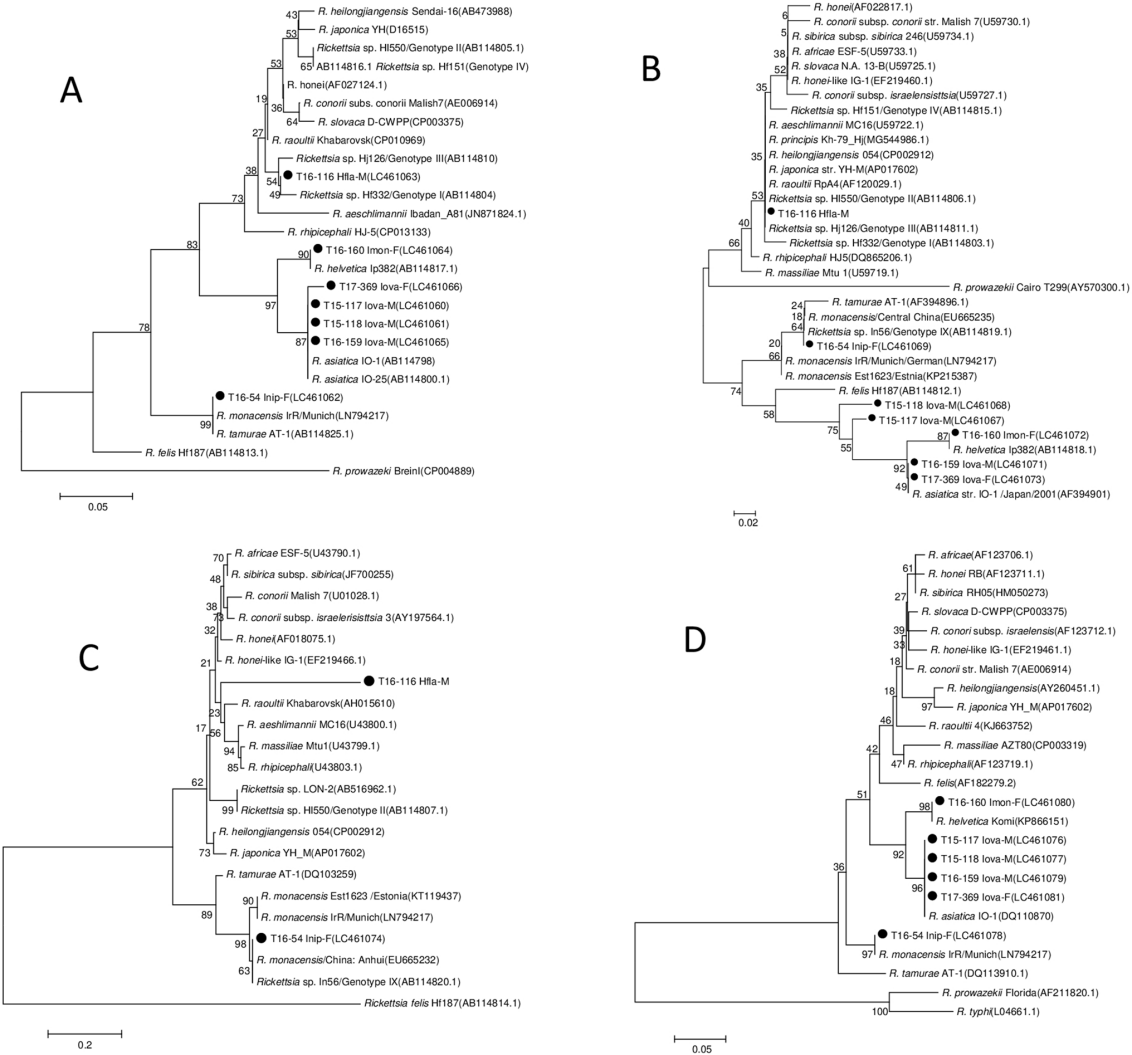
Phylogenetic analysis for identification of species of *Rickettsiae* based on 450 nucleotides of 17 kDa gene **(A)**, 382 nucleotides of *gltA* gene **(B)**, 540 nucleotides of rO*mpA* gene **(C)** and 426 nucleotides of rO*mpB* gene **(D)**. Sequence were aligned by using MEGA5 software (https://www.megasoftware.net). Neighbor-joining phylogenetic tree construction and bootstrap analysis were performed according to the Kimura 2-parameter distances method. Bold-face font indicate positive samples detected from ticks in this study (shown only representative sample no. from among detected SFGR).

All the 1373 PCR amplicons of SFGR 17-kDa (410 bp) and *gltA* (342 bp) were sequenced, and none of them presented 100% identity with *R. japonica*. PCR targeting and sequencing of *rOmpA* (540 bp) and *rOmpB* (381 bp) were conducted with the DNA samples of adult ticks, then the *Rickettsia* spp. were identified using sequence data of 17-kDa, *gltA*, *rOmpA* and *rOmpB* (Fig. 2, Table 4).

**Table 4:** Confirmed species/genus of SFGR in this study by sequencing.

Sample no	Tick			Amplification of gene		Species of *Rickettsiae*	Sequence of gene
	Collection site	Species	Stage/sex	*rOmpA*	*rOmpB*		
T16-159	No. 9	*I. ovatus*	Adult/M	No	Yes	*R. asiatica*	
T17-79	No. 13	*I. ovatus*	Adult/F	No	Yes	*R. asiatica*	Same as T16-159
T17-81	No. 13	*I. ovatus*	Adult/F	No	Yes	*R. asiatica*	Same as T16-159
T17-82	No. 13	*I. ovatus*	Adult/F	No	Yes	*R. asiatica*	Same as T16-159
T17-702	No. 13	*I. ovatus*	Adult/F	No	Yes	*R. asiatica*	Same as T16-159
T17-80	No. 13	*I. ovatus*	Adult/M	No	Yes	*R. asiatica*	Same as T16-159
T17-418	No. 16	*I. ovatus*	Adult/F	No	Yes	*R. asiatica*	Same as T16-159
T17-421	No. 16	*I. ovatus*	Adult/F	No	Yes	*R. asiatica*	Same as T16-159
T17-402	No. 16	*I. ovatus*	Adult/M	No	Yes	*R. asiatica*	Same as T16-159
T17-411	No. 16	*I. ovatus*	Adult/M	No	Yes	*R. asiatica*	Same as T16-159
T17-442	No. 17	*I. ovatus*	Adult/M	No	Yes	*R. asiatica*	Same as T16-159
T17-369	No. 18	*I. ovatus*	Adult/F	No	Yes	*R. asiatica*	
T17-371	No. 18	*I. ovatus*	Adult/F	No	Yes	*R. asiatica*	Same as T16-159
T17-111	No. 28	*I. ovatus*	Adult/F	No	Yes	*R. asiatica*	Same as T16-159
T17-112	No. 28	*I. ovatus*	Adult/F	No	Yes	*R. asiatica*	Same as T16-159
T15-117	No. 28	*I. ovatus*	Adult/M	No	Yes	*R. asiatica*	
T15-118	No. 28	*I. ovatus*	Adult/M	No	Yes	*R. asiatica*	
T17-109	No. 28	*I. ovatus*	Adult/M	No	Yes	*R. asiatica*	Same as T16-159
T17-163	No. 29	*I. ovatus*	Adult/M	No	Yes	*R. asiatica*	Same as T17-369
T17-212	No. 30	*I. ovatus*	Adult/F	No	Yes	*R. asiatica*	Same as T16-159
T17-269	No. 30	*I. ovatus*	Adult/F	No	Yes	*R. asiatica*	Same as T16-159
T17-259	No. 30	*I. ovatus*	Adult/M	No	Yes	*R. asiatica*	Same as T16-159
T17-510	No. 32	*I. ovatus*	Adult/F	No	Yes	*R. asiatica*	Same as T16-159
T17-519	No. 32	*I. ovatus*	Adult/F	No	Yes	*R. asiatica*	Same as T16-159
T17-507	No. 32	*I. ovatus*	Adult/M	No	Yes	*R. asiatica*	Same as T16-159
T17-508	No. 32	*I. ovatus*	Adult/M	No	Yes	*R. asiatica*	Same as T16-159
T17-477	No. 37	*I. ovatus*	Adult/F	No	Yes	*R. asiatica*	Same as T16-159
T17-473	No. 38	*I. ovatus*	Adult/F	No	Yes	*R. asiatica*	Same as T16-159
T17-474	No. 38	*I. ovatus*	Adult/F	No	Yes	*R. asiatica*	Same as T16-159
T16-174	No. 6	*I. monospinosus*	Adult/F	No	Yes	*R. helvetica*	Same as T16-160
T16-160	No. 9	*I. monospinosus*	Adult/F	No	Yes	*R. helvetica*	
T16-145	No. 10	*I. monospinosus*	Adult/F	No	Yes	*R. helvetica*	Same as T16-160
T16-146	No. 10	*I. monospinosus*	Adult/M	No	Yes	*R. helvetica*	Same as T16-160
T16-186	No. 6	*I. nipponensis*	Adult/M	Yes	Yes	*R. monacensis*	Same as T16-54
T16-122	No. 13	*I. nipponensis*	Adult/M	Yes	Yes	*R. monacensis*	Same as T16-54
T16-228	No. 15	*I. nipponensis*	Adult/M	Yes	Yes	*R. monacensis*	Same as T16-54
T17-424	No. 16	*I. nipponensis*	Adult/M	Yes	Yes	*R. monacensis*	Same as T16-54
T16- 54	No. 28	*I. nipponensis*	Adult/F	Yes	Yes	*R. monacensis*	
T17-525	No. 32	*I. nipponensis*	Adult/F	Yes	Yes	*R. monacensis*	Same as T16-54
T17-524	No. 32	*I. nipponensis*	Adult/M	Yes	Yes	*R. monacensis*	Same as T16-54
T16-116	No. 13	*H. flava*	Adult/M	Yes	No	*Rickettsia* sp.	
T16-209	No. 14	*H. flava*	Adult/M	Yes	No	*Rickettsia* sp.	Same as T16-116

A total of 25 out of 29 (89.6%) SFGR positive samples, including sample T16-159 (GenBank accession no. LC461065, LC461071 and LC461079) detected in *I. ovatus* presented 100% identity with *Rickettsia asiatica* IO-1 (AB114798, AF394901 and DQ113910) in 17-kDa, *gltA* and *rOmpB* sequences. None of the 29 samples amplified *rOmpA* in PCR. For sample T17-369 (LC461066, LC461073 and LC461081) and T17-163, *gltA* and *rOmpB* sequences yielded 100% identity with *R. asiatica* IO-1 and 99.8% identity for the 17-kDa. For the sample T15-117 (LC4610461060, LC461067 and LC461076) and T15-118 (LC461061, LC461068 and LC461077), 17 kDa and *rOmpB* amplicons were 100% identical to *R. asiatica* IO-1 whereas the sequences of *gltA* presented 99.4% and 98.8% identity for T15-117 and T15-118, respectively.

**Figure 3:**
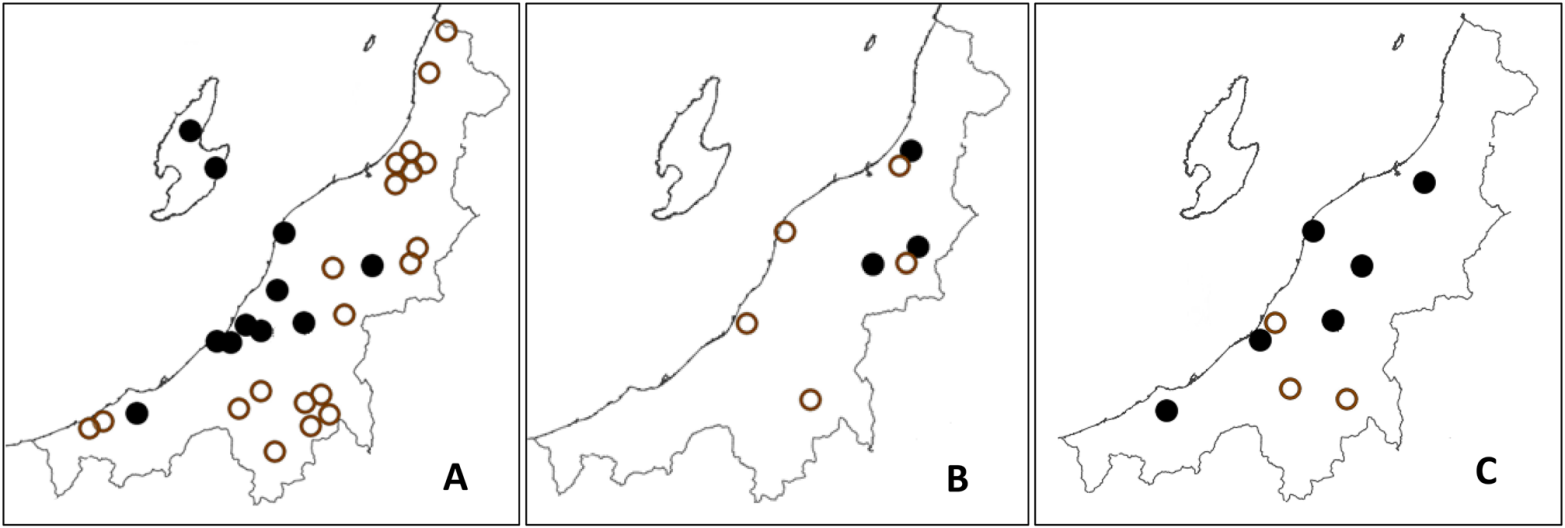
Niigata prefecture map with the sites where adult stages of *Ixodes* spp. were collected (Circles), and the occurrence of SFGR (filled circle: SFGR Positive sites, open circle: SFGR Negative sites). From the left, “A” corresponds to *I. ovatus* and *Rickettsia asiatica* sites; “B” corresponds to *I. monospinosus* and *R. helvetica* sites, and “C” are the *I. nipponensis* and *R. monacensis* sites. Maps created using the Geographical Survey Institute Map Vector from the Geospatial Information Authority of Japan (https://maps.gsi.go.jp/).

*I. ovatus* was collected in 31 sites, though *R. asiatica* was detected from *I. ovatus* collected in 11 of these sites (Table 5, Fig. 3A). *I. ovatus* harboring *R. asiatica* were collected in the central and western part of the prefecture and Sado Island, but not from northeast and mountainous area of the prefectural border with Gunma (collection sites Nos. 20–22, 24–27). Overall, *R. asiatica* was detected in 8.4% of the *I. ovatus* adult samples; however, the infection rate varied by collection site such as in Sado Island (site No. 37 and 38 in total) with an infection rate of 50%, Mt. Kakuda (Site No. 13) with 36%, and in Satogaike Sports Field (Site No. 28) with 26%.

**Table 5:** Prevalence of rickettsial genes detected from adult ticks by collection sites. The Northern (Kaetsu), Central (Chuetsu) and Southern (Joetsu) areas of Niigata Prefecture corresponds to the collection sites 1 to 13, 14 to 30, and 31 to 36, respectively. Sado Island area corresponds to the collection sites 37 and 38.

Species of tick	Site no																																						Total
	1	2	3	4	5	6	7	8	9	10	11	12	13	14	15	16	17	18	19	20	21	22	23	24	25	26	27	28	29	30	31	32	33	34	35	36	37	38	
*I. monospinosus*	–	–	–	0/1	–	1/2	–	–	1/1	2/2	0/2	–	0/2	–	–	–	–	–	–	–	0/1	–	–	–	–	–	–	–	–	0/1	–	–	–	–	–	–	–	–	4/12*
*I. nipponensis*	–	–	–	–	–	1/2	–	–	–	–	–	–	1/1	–	1/2	1/1	–	–	–	0/1	–	–	–	–	–	0/1	–	1/2	–	0/2	–	2/4	–	–	–	–	–	–	7/16
*I. ovatus*	0/4	0/3	–	0/9	0/5	0/11	0/4	0/4	1/4	0/2	0/7	–	5/14	0/23	0/5	4/23	1/14	2/34	–	0/15	0/3	0/5	–	0/2	0/11	0/32	0/1	5/19	1/6	3/28	–	4/33	0/6	0/11	–	–	1/4	2/2	29/344

*I. monospinosus* was present in eight sites. From three sites (site No. 6, 9 and 10) in the northeast region of the prefecture, 4 out of 5 *I. monospinosus* presented *Rickettsia helvetica*. In the other five sites with *I. monospinosus*, *R. helvetica* was not found (Table 5, Fig. 3B). The four SFGR positive samples including sample T16-160 (LC461064, LC461072 and LC461080) from *I. monospinosus* adult ticks had 100% identity with *Rickettsia helvetica* IP382 (AB114817) in 17 kDa, *gltA* and *rOmpB* amplicons. None of the four samples amplified *rOmpA* in PCR.

From seven SFGR positive samples, including sample T16-54 (LC461062, LC461069, LC461074 and LC461078), which were detected in *I. nipponensis* adult ticks had 100% identity with *Rickettsia monacensis* IrR Munich (LN794217) in 17 kDa and *rOmpB* amplicons. The amplicons of 17 kDa showed 100% identity with *Rickettsia tamurae* AT-1 (AF394896); however, the similarity of the *rOmpB* amplicons was 97.4% with *R. tamurae* AT-1 (AF394896). For *gltA* amplicons, the similarities were 99.7% with *R. monacensis* IrR Munich (LN794217) and 99.4% with *R. tamurae* AT-1 (AF394896). For *rOmpA* amplicons, the similarities were 99.3% with *R. monacensis* IrR Munich (LN794217) and 95.4% with *R. tamurae* AT-1 (AF394896). Obtained sequences in this study produced a different cluster from *R. tamurae* in *gltA, rOmpA,* and *rOmpB* regions; therefore, the seven SFGR samples including T16-54 detected in *I. nipponensis* adult ticks were concluded as *R. monacensis*. In *Ixodes* spp., the presence of *R. monacensis* was high as 43.8% in *I. nipponensis*, in ticks collected in 6 out of 9 sites (Table 5, Fig. 3C).

Two SFGR samples, including sample T16-116 (LC461063, LC461070, and LC461075), detected in *H. flava* adult presented the same sequences in 17 kDa, *gltA,* and *rOmpA* regions. Both of them did not amplify in *rOmpB* PCR. The similarity in the 17 kDa region was 99.8% with *Rickettsia* sp. Hf332 (AB114804) and *Rickettsia* sp. Hj126 (AB114810), and for *gltA*, the similarities were 99.7% with *Rickettsia* sp. Hf332 (AB114804) and 100% with *Rickettsia* sp. Hj126 (AB114810). For the *rOmpA* gene, there were no matched sequences in GenBank.

## Discussion

The last tick survey in Niigata Prefecture was done in the ’50s^6^. In this study, we collected *D. taiwanensis, H. hystricis, H. megaspinosa, I. columnae,* and *I. monospinosus* species not observed in the previous study, showing the presence of ticks may have been influenced by the environmental change and hosts movement (Sato et al., in preparation). This new ticks/host distribution pattern could bring the pathogen near to humans, facilitating the infection by tick-borne pathogens^15^. SFGR was detected in ticks collected in 20 of 38 sites from all the collection sites in Niigata Prefecture. In 16 of 19 sites where SFGR positive ticks were not collected, there was a low number of collected ticks (lower than 20), and it might have influenced the SFGR detection rates, as seen in the low prevalence of the SFGR in ticks. To understand the SFGR prevalence in the prefecture, continuous tick collection is needed, especially in sites where the collection number is low. SFGR positivity in adult ticks in Niigata Prefecture was 5.6%, and it is similar to the positivity rate of the neighboring prefecture, Toyama, with 3.3%^15^. However, when the SFGR detection rate is compared to other prefectures, such as Fukui (22.0%) and six western prefectures including Shizuoka (21.6%)^16,17^, the SFGR positivity in Niigata Prefecture is still low. In the western part of Japan, SFGR positivity was reported to be as high as 40.5% in *H. longicornis*^17^; in contrast, in hokuriku region of Honshu (incl. Niigata, Toyama, Ishikawa, and Fukui Prefectures), SFGR positivity rates are high in *I. monospinosus*, with 50% in Toyama^15^, 43.8% in Fukui^16^ and 43.8% in Niigata (this study). The tick species prevalence depends on the area/region, therefore the prevalence of the SFGR, and *Rickettsia* spp. could also vary. *Rickettsia* spp. have strong host-specificity^15–17^ and, SFGR detected in this study confirmed this feature. Ticks and *Rickettsia* sp. were: *R. asiatica* from *I. ovatus*, *R.helvetica* from *I. monospinosus,* and *R. monacensis* from *I. nipponensis*.

The first report of *R. asiatica* was in Fukushima Prefecture in 1993, described as *Rickettsia* sp. IO-1 in *I. ovatus* with subsequent reports in other areas^18,19^. Moreover, *R. asiatica* was detected in other tick species, such as *H. flava*, *H. japonica,* and *H. hystricis* ^9^, showing a diverse ticks host preference. Regarding mammalian hosts, *R. asiatica* was detected in blood samples of Japanese deer (*Cervus nippon*); however, the pathogenicity in these hosts is unknown^20^. SFGR detection in *I. ovatus* in the neighboring prefecture is varied, with rates of 0.0% in Toyama^15^, 7.9% in Fukui^16^, and 8.4% in Niigata (this study). Also, in this case, the positivity rates may vary according to the number of sampling sites and sampling size. It is not clear if the *R. asiatica* positivity is influenced by the ecology of *I. ovatus*, environmental factors, or ticks’ susceptibility for pathogens. Continuous research is needed including studies on environmental change and ticks endemicity.

*R. helvetica* was reported as Japanese spotted fever pathogen in Fukui Prefecture ^21^, and it is also detected in *I. ovatus, I. persulcatus,* and *H. japonica* ^19^. In this study, *R. helvetica* was detected only from *I. monospinosus*, with a positivity rate of 33.3%. In Toyama Prefecture, *R. helvetica* was detected from 2 of 4 *I. monospinosus* ^15^. In this study, *R. helvetica* positive *I. monospinosus* was present only in the northeast area of Niigata Prefecture (Site No 6, 9 and 10) (Fig. 3B); however, there was a limited number of *I. monospinosus* adults (N = 12), present in 8 out of 38 collection sites. To confirm these host specificity and region preferences, further tick collection and field surveys are necessary.

There is only one report of SFGR detected from *I. nipponensis* in the Toyama prefectural area, reported as *Rickettsia* sp. In56 ^9^. In this study, seven samples were positives to SFGR in *I. nipponensis* with 100% identity with *Rickettsia* sp. In56 (AB114819, AB114820) in *gltA* and *rOmpA* regions. Therefore *Rickettsia* sp. In56 might be *R. monacensis*. In Europe, *R. monacensis* is indicated as a spotted fever pathogen^22,23^ and was also isolated from a spotted fever patient in Korea^24^. The tick species harboring *R. monacensis* is *Ixodes ricinus* in Europe, and in China, the same pathogen was described in *I. persulcatus* and *Ixodes sinensis* ^25–27^. In Korea, similar to this study, *R. monacensis* was detected from *I. nipponensis*^28^. In this study, *I. nipponensis* presented the highest SFGR positivity in all the collected tick species; SFGR positive *I. nipponensis* were found from 7 out of 10 sites, indicating *R. monacensis* might be widely prevalent in Niigata Prefecture.

In *Rickettsia* sp. Hj126 (AB114803) and *Candidatus* Rickettsia principis Kh-79_Hj (MG544986), 2 SFGR detected in adult *H. flava,* the *gltA* region presented 100% identity and were classified as genotype III by Ishikura’s categorization^9^ (Fig. 2). The SFGR of the genotype III detected in Japan^9^, presented the same characteristics of the two SFGR detected in this study, indicating SFGR of the genotype III might be widely prevalent in Japan.

In this study, *R. japonica* was not detected in the ticks, despite having a case of Japanese spotted fever in 2014, and *D. taiwanensis* and *H. hystricis* which are known vectors for *R. japonica*^29^ were collected. More wide sampling and/or larger sample size could be necessary to detect a low prevalent species in the arthropod hosts. Additionally, from a clinical point of view, the implementation of serology and DNA isolation might improve the diagnosis and management of patients with spotted fever like illnesses, as recommended in Europe in case of Mediterranean spotted fever like patients^30^.

Three causative agents of human spotted fever *R. asiatica*, *R. helvetica,* and *R. monacensis*, were detected in this study. The major SFGR positive ticks were *Ixodes* spp. followed by *Haemaphysalis* spp. High tick-pathogen specificity was also observed in *Ixodes* sp. and *Rickettsia* sp.

Continuous precaution is recommended in activities where there is a potential risk of contact with ticks, and the healthcare system should be aware of spotted fever, particularly since Niigata Prefecture can now be considered an SFGR endemic area and human cases may be occurring.

## Data Availability

All data generated or analysed during this study are included in this published article.
